# Applying implementation science to improve care for familial hypercholesterolemia

**DOI:** 10.1097/MED.0000000000000692

**Published:** 2021-12-09

**Authors:** Laney K. Jones, Ross C. Brownson, Marc S. Williams

**Affiliations:** a Genomic Medicine Institute, Geisinger, Danville, PA, USA; b Department of Surgery (Division of Public Health Sciences) and Siteman Cancer Center, Washington University School of Medicine, Washington University in St. Louis, St. Louis, Missouri, USA; c Prevention Research Center in St. Louis, Brown School, Washington University in St. Louis, St. Louis, Missouri, USA

**Keywords:** cascade screening, familial hypercholesterolemia, identification, implementation science

## Abstract

Improving care of individuals with familial hypercholesteremia (FH) is reliant on the synthesis of evidence-based guidelines and their subsequent implementation into clinical care. This review describes implementation strategies, defined as methods to improve translation of evidence into FH care, that have been mapped to strategies from the Expert Recommendations for Implementing Change (ERIC) compilation.

**Recent findings:**

A search using the term ‘familial hypercholesterolemia’ returned 1350 articles from November 2018 to July 2021. Among these, there were 153 articles related to improving FH care; 1156 were excluded and the remaining 37 were mapped to the ERIC compilation of strategies: assess for readiness and identify barriers and facilitators [9], develop and organize quality monitoring systems [14], create new clinical teams [2], facilitate relay of clinical data to providers [4], and involve patients and family members [8]. There were only 8 of 37 studies that utilized an implementation science theory, model, or framework and two that explicitly addressed health disparities or equity.

**Summary:**

The mapping of the studies to implementation strategies from the ERIC compilation provides a framework for organizing current strategies to improve FH care. This study identifies potential areas for the development of implementation strategies to target unaddressed aspects of FH care.

## INTRODUCTION

**FIGURE 1:**
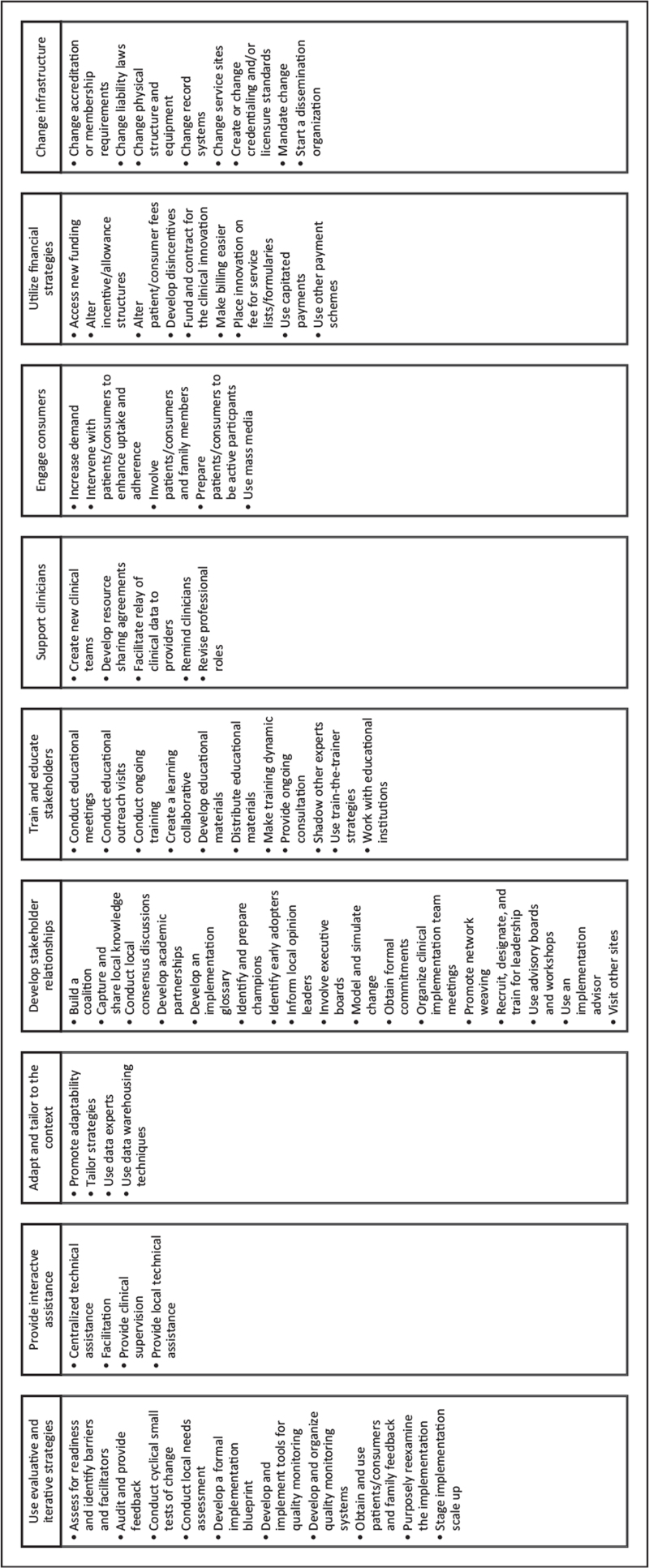
List of the 73 Expert Recommendations for Implementing Change (ERIC) implementation strategies categorized by nine overarching themes.

Improving care of individuals with familial hypercholesteremia (FH) is reliant on the synthesis of evidence-based guidelines and their subsequent implementation into clinical care. Recent Cholesterol Guidelines provide evidence-based clinical guidance for caring for individuals with FH [1,2]. However, not all these recommendations have been implemented into clinical care (e.g., systematic identification of individuals with FH [3]). The field of implementation science supplies theories, models, and frameworks for the development and implementation of strategies to reduce the time from discovery to translation into clinical practice [4,5]. Compilations of implementation strategies, defined as ‘methods or techniques used to enhance the adoption, implementation, and sustainability of a clinical program or practice’ [6], have been developed, such as the Expert Recommendation for Implementation Change (ERIC) [7] and Effective Practice and Organization of Care (EPOC) [8]. The purpose of ERIC and EPOC was to develop a list of commonly used implementation strategies and then to create a standard naming schema for those strategies accompanied by standardized definitions that could be modified for specific studies. Figure 1 provides a list of 73 ERIC strategies categorized into nine overarching themes. This review describes implementation strategies, defined as methods to improve translation of evidence into FH care, that have been mapped to standardized compilation of strategies. 

**Box 1:**
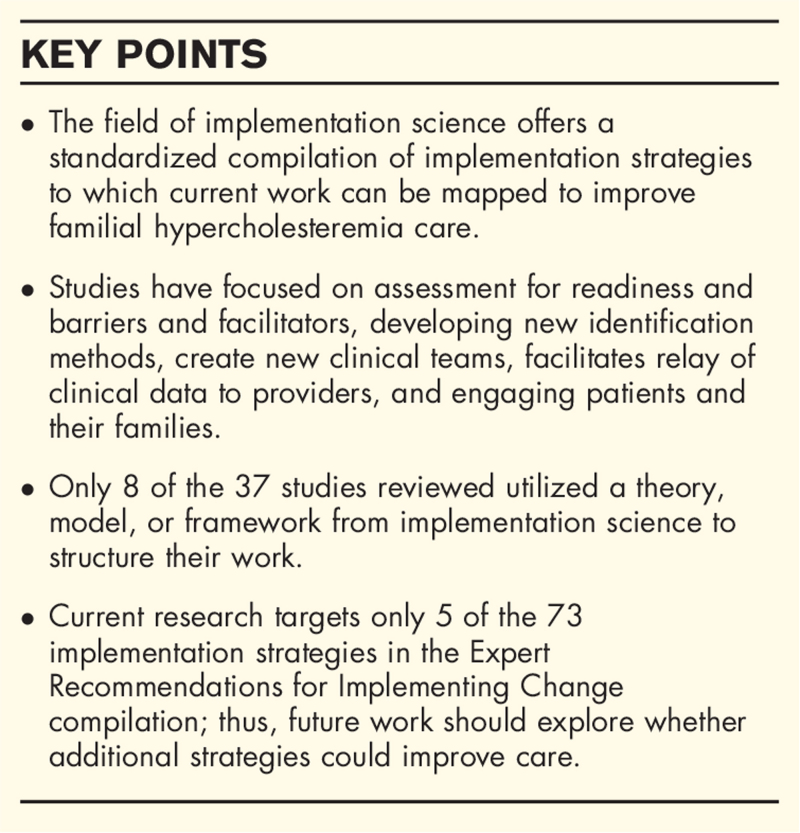
no caption available

## METHODS

**FIGURE 2:**
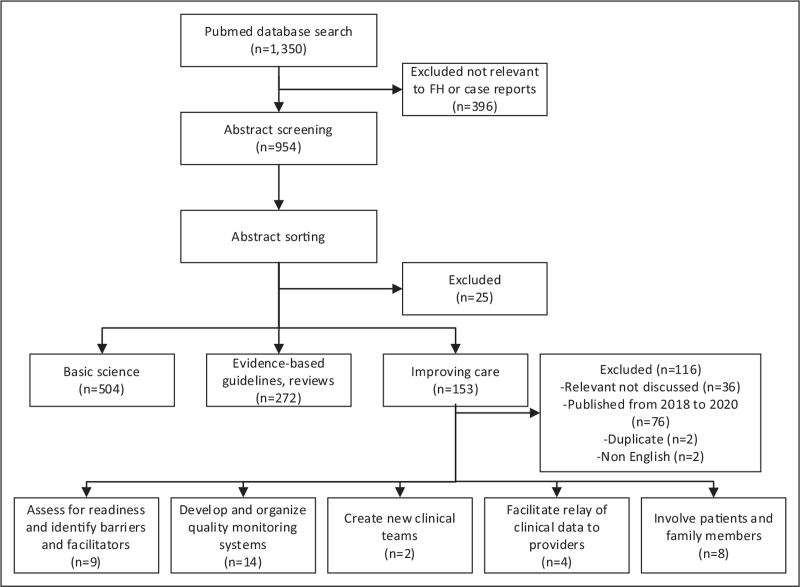
Flow diagram of articles included in the review.

We conducted a scoping review of the literature focused on studies to improve care for individuals with FH [9]. We searched PubMed from November 1, 2018 to July 31, 2021 to identify all relevant articles that were published after the release of the 2018 AHA/ACC Multi-Society Cholesterol Guidelines [1]. This search returned 1350 articles when using key words associated with ‘familial hypercholesterolemia’ (PubMed search strategy Table 1). During phase 1 of abstract screening, studies were excluded that were case reports, or articles not relevant to FH. During phase 2 of abstract screening, studies were sorted into three categories: findings in basic science (i.e., discovery), evidence-based guidelines/reviews, and suggestions for improving care for individuals with FH. All abstract screening was completed by a single reviewer. The focus of this review was only articles in the latter category which included any studies that explored aspects related to implementation of an evidence-based intervention for adults. Included full text articles were categorized into one of the 73 implementation strategies from the ERIC compilation. The ERIC compilation was selected as the standardized list of implementation strategies because the identified strategies in the articles reviewed better aligned with this compilation. Figure 2 depicts the article review process and categorization. Each article was coded if they utilized an implementation science theory, model, or framework or focused on health disparities or equity by identifying barriers to care or strategies to reduce care variation in certain populations.

**Table 1:** PubMed search strategy

PubMed search strategy
“Hyperlipoproteinemia Type II’[Mesh] OR “familial hypercholesterolaemia’[All Fields] OR “hyperlipoproteinemia type ii’[Mesh] OR (“hyperlipoproteinemia’[All Fields] AND “type’[All Fields] AND “ii’[All Fields]) OR “hyperlipoproteinemia type ii’[All Fields] OR (“familial’[All Fields] AND “hypercholesterolemia’[All Fields]) OR “familial hypercholesterolemia’[All Fields].

## RESULTS

Of the 1350 articles found, 954 abstracts were sorted into three categories: basic science (*n* = 504), evidence-based guidelines/reviews (*n* = 272) and improving care (*n* = 153). Of the 153 articles in the improving care category, 116 were excluded, as they were relevant but either did not map to implementation strategies, focused on pediatric care, published between 2018 and 2020, duplicates, or not available in English. The remaining 37 were categorized into the following implementation strategies: ‘assess for readiness and identify barriers and facilitators’ [9], ‘develop and organize quality monitoring systems’ [14], ‘create new clinical teams’ [2], ‘facilitate relay of clinical data to providers’ [4], and ‘involve patients and family members’ [8]. Table 2 lists and defines the mapped ERIC implementation strategies. There were only 8 of 37 studies that utilized an implementation science theory, model, or framework (three of the eight were published by the first author of this manuscript) and two that explicitly addressed health disparities or equity. Table 3 details the studies included in the review categorized by the ERIC compilation of strategies and coded for including an implementation science theory, model, or framework and mention of health disparities or equity.

**Table 2:** Categorization and definitions of implementation strategies to the Expert Recommendations for Implementing Change (ERIC) compilation

Implementation strategy	Number of studies	Definition
Develop and organize quality monitoring systems	14	Develop and improve diagnostic performance of tools to identify individuals with FH
Assess for readiness and identify barriers and facilitators	9	Assess healthcare organizations and providers to determine their degree of readiness to implement and barriers and enablers to FH care
Involved patients/consumers and family members	8	Engage or include patients and families to improve FH care
Create new clinical teams	2	Change who serves on the clinical team, adding different disciplines, and different skills to the FH care team
Facilitate relay of clinical data to providers	4	Provide data using integrated modes of communication to improve FH care

FH, familial hypercholesteremia.

**Table 3:** Description of studies included in the review

Study	Year	Design	Country	Implementation strategy	Implementation science theory, model, framework	Health disparities or equity focus^a^
Assess for readiness and identify barriers and facilitators (*n* = 9)						
Jones *et al.*	2021	Qualitative analysis	United States	Focus groups with individuals with FH and providers on the acceptability, appropriateness, and feasibility of identification and cascade screening methods for FH	Conceptual Model of Implementation Research	
Jones *et al.*	2020	Qualitative analysis	United States	Interviews and focus groups with individuals with FH and providers to discuss barriers and facilitators and develop potential solutions to improve treatment approaches	Practical, Robust Implementation and Sustainability Model	
Kawasaki *et al.*	2021	Prepost	Japan	Genetic literacy education program for providers		
Miller *et al.*	2021	Qualitative analysis	United States	Interviews with key informants regarding barriers and recommendations to improve FH screening	Reach, Effectiveness, Adoption, Implementation, and Maintenance	
Mszar *et al.*	2021	Cross sectional	United States	Survey based on the health belief model to understand self-efficacy, perceived barriers to care and health-promoting behaviors across cardiovascular risk factors	Health Belief Model	Yes
Schwiter *et al.*	2020	Cross sectional	United States, International	Survey of perspectives regarding direct contact as an approach for cascade screening of relatives		
Wand *et al.*	2020	Cross sectional	United States	Survey of clinically diagnosed FH patients regarding intention to obtain genetic testing		
Wong *et al.*	2021	Cross sectional	United States	Survey of primary care physicians and cardiologists regarding perceptions and barriers to use of PCSK9 inhibitors in FH		
Unim *et al.*	2020	Cross sectional	Canada	Survey of healthcare workers on barriers to genetic testing		
Develop and organize quality monitoring systems (*n* = 14)						
Abul-Husn *et al.*	2021	Cross sectional	United States	Population genetic screening		Yes
Akyea *et al.*	2020	Cross sectional	United Kingdom	EHR data screening tool (FAMCAT)		
Akyea *et al.*	2020	Diagnostic accuracy	United Kingdom	Machine learning algorithm		
Birnbaum *et al.*	2021	Prospective cohort	United States	EHR data screening tool (MEDPED primary)		
Buchanan *et al.*	2020	Cross sectional	United States	Population genetic screening		
David *et al.*	2021	Cross sectional	United States	Population genetic screening		
Ingoe *et al.*	2021	Cross sectional	United Kingdom	EHR data screening tool (Simon Broome primary)		
Grzymski *et al.*	2020	Cross sectional	United States	Population genetic screening		
Kawame *et al.*	2021	Noncontrolled	Japan	Population genetic screening		
Pepplinkhuizen *et al.*	2020	Cross sectional	Netherlands	EHR data screening tool (DLCN primary)		
Pina *et al.*	2020	Diagnostic accuracy	Sweden and Italy	Machine learning algorithm (compared to DLCN)		
Sabatel-Perez *et al.*	2021	Cross sectional	Spain	EHR data screening tool (DLCN primary)		
Sheth *et al.*	2021	Cross sectional	United States	Machine learning algorithm		
Zamora *et al.*	2021	Cross sectional	Spain	EHR data screening tool (7 different phenotype algorithms were tested)		
Create new clinical teams (*n* = 2)						
Jones *et al.*	2021	Cross sectional	United States	Implementation and evaluation of a multidisciplinary lipid clinic	Reach, effectiveness, adoption, implementation, and maintenance	
Wilkinson *et al.*	2020	Cross sectional	United Kingdom	Implementation and evaluation of a nurse-led lipid clinic		
Facilitate relay of clinical data to providers (*n* = 4)						
Bangash *et al.*	2020	Qualitative analysis	United States	Interview and survey with providers for development and implementation of a CDS tool	Conceptual Framework of Implementation Research	
Ellis *et al.*	2020	Cross sectional	Australia	Impact of genetic risk scores		
Gallo *et al.*	2021	Cross sectional	France	Contribution of coronary calcium scores to SAFEHEART-RE		
Ramos *et al.*	2020	Cross sectional	Spain	Performance of the SIDIAP-FHP score compared to SAFEHEART-RE		
Involved patients and family members (*n* = 8)						
Baldry *et al.*	2021	Prepost	United States	Motivational interviewing and extended parallel process model		
Benatar *et al.*	2020	Qualitative	New Zealand	Family visit with healthcare professionals and initiation of a family Facebook^®^ page to discuss family implications of an FH result		
Descamps *et al.*	2020	Cross sectional	Belgium	Probands were screened by specialist and met DLCN score ≥6 and then relatives were visited for screening		
Gidding *et al.*	2020	Cross sectional	United States	Individuals were recruited from the FH CASACDE^®^ Registry to undergo genetic testing and their first-degree relatives could also receive testing		
Kinnear *et al.*	2020	Qualitative analysis	United Kingdom	Theory informed behavior change intervention to improve adherence to dietary and physical activity guidelines for individuals with FH	Behavior change wheel and Theoretical domains framework	
Kinnear *et al.*	2020	Cross sectional	United Kingdom	Results of feasibility trial of the intervention to improve adherence to dietary and physical activity guidelines	Behavior change wheel and Theoretical domains framework	
McGowan *et al.*	2021	Prepost	United States	FH Foundation directly engaged with FH probands and relatives		
Neuner *et al.*	2020	Cross sectional	United States	Probands were identified via web-based risk assessment service (MeTree) linked to EHR information or EHR query alone, if positive, relatives were invited to receive genetic testing		

CDS, clinical decision support; DLCN, Dutch Lipid Clinic Network criteria; EHR, electronic health records; FH, familial hypercholesteremia; MEDPED, Make Early Diagnosis to Prevent Early Deaths; SAFEHEART-RE, Spanish FH Cohort Study risk equation.

a Focus on health disparities or equity by identifying barriers to care or strategies to reduce care variation in certain populations.

### Assess for readiness and identify barriers and facilitators

Lack of a systematic and sustainable approach to identifying individuals with FH leads to delays in care [10]. A survey of providers found significant barriers to providers offering genetic testing to their patients and barriers that providers perceived patients having to the acceptability of genetic testing including limited coverage by insurance companies, availability of personnel to explain and order testing, and lack of access to genetic counseling professionals [11]. However, when individuals with a clinical diagnosis of FH were surveyed three factors were associated with their willingness to undergo genetic testing. These factors included aversion to FH genetic information, curiosity regarding medical and family history, psychological reassurance of genetic testing intent [12].

These barriers identified by providers and patients have led researchers to develop educational strategies to improve uptake of genetic testing. The implications of a genetic literacy program to address these barriers found that providers improved their understanding about genetics and ability to provide accurate knowledge and advice while promoting genetic literacy to patients [13]. Similarly, for cascade screening of relatives, an international survey explored perspectives of patients on indirect and direct contact approaches for cascade screening and found that a majority of individuals supported direct outreach by their provider to their relatives to share their FH result [14]; however, this approach is seldom used.

Barriers and facilitators to improving access to care for FH [15^▪▪^] and treatment approaches for FH exist [16^▪▪^]. Articles included share similar findings: awareness of FH is poor, guidelines are complex and changing, and a focused supportive effort is needed to improve FH management [15^▪▪^,^16^^▪▪^]. A recent study found 30% of young patients with FH had poor adherence to lipid-lowering therapies, the main reason being lack of motivation. A survey of primary care physicians and cardiologists found several factors influencing prescribing of PCSK9 inhibitors: clinical type (cardiologist more likely to order) and practice setting and location (urban and academic centers more likely to order) [17].

Assessment of stakeholder readiness to implement is important for successful uptake of an evidence-based intervention [18^▪▪^,^19^^▪▪^]. Focus groups with stakeholders that addressed willingness to use novel identification processes including automated approaches (i.e., machine learning) and cascade screening methods for FH, including chatbots and direct contact. They found these methods were acceptable, appropriate, and feasible if they fit into the clinician workflow [19^▪▪^].

### Develop and organize quality monitoring systems

Four studies implemented the existing clinical diagnostic criteria into their healthcare system electronic health records (EHRs) as a screening tool to identify previously unrecognized individuals with FH. Similar rates of individuals requiring additional diagnostic screening for FH were found: 1 in 245 (7468/1 831 658) met the Make Early Diagnoses Prevent Early Deaths (MEDPED) criteria [20], 1 in 150 (303/45 123) met the Simon Broome (SB) Criteria [21], and 1 in 183 (269/49 321) [21] and 1 in 119 (351/41 937) [22] met the Dutch Lipid Clinic Network Criteria (DLCN). The screening positive rate for FH was higher, 1 in 5 (84/469), when the DLCN criteria were applied to EHRs of those with known severe hypercholesterolemia [23]. Diagnostic evaluation for FH in individuals identified by these EHR screening initiatives found 18–36% met clinical criteria [21–23]. However, the percentage of these individuals with a genomic risk variant for FH ranged from 25 to 68% depending on the study [20,21,23] meaning that using genetics as the sole indicator for a diagnosis of FH would miss many individuals who met clinical diagnostic criteria.

Instead of utilizing the traditional clinical diagnostic criteria, some have implemented specific algorithms that use clinical data available in the EHR [24,25]. The most efficient of the seven algorithms tested that could be translated into clinical practice identified 840 patients with FH [24]. Another study found the FH case ascertainment identification tool (FAMCAT) algorithm to have a high level of discrimination (area under the curve [AUC] = 0.844, 95% confidence interval [CI] = 0.834–0.854) and performed better when compared to the manual scoring of the SB criteria (AUC = 0.730, 95% CI = 0.719 to 0.741) and DLCN Score (AUC = 0.766, 95% CI = 0.755 to 0.778) [25].

The use of machine learning approaches to identify individuals with FH is novel and positive results from these studies provide insight into their capabilities to help close the FH identification gap [26,27]. A machine learning algorithm that utilized five different approaches (logistic regression, random forest, gradient boosting machines, neural networks, and ensemble learning) had high predictive accuracy (AUC > 0.89) [26]. Three machine learning algorithm approaches (classification tree, gradient boosting machine, and neural network) were found to perform better than applying the DLCN criteria alone [27]. There is still more to learn on how to successfully move from identification approaches to implementation into clinical care. A study utilizing the FH Foundation's FIND FH machine learning algorithm (random forest) identified 5006 screened positive patients but only 153 were seen for clinical confirmation [28]. Implementation at the healthcare system level will be required to fully realize the potential of information-technology based tools.

Five healthcare systems have implemented population genetic screening approaches to identify unselected individuals with risk for genetic disease including Tier 1 genetic conditions (designated by the Centers for Disease Control and Prevention's Office of Public Health Genomics [29]) including FH [30^▪^,^31^–^34^]. Each of these population screening approaches performs exome sequencing, links exome data to EHR systems, returns actionable results, and allows for recontact for future studies. To date, these programs have identified participants with variants in three genes associated with FH (*LDLR, APOB, PCSK9*). Rates of identification: Mt. Sinai 8 in 692, Geisinger 93 in 64 392, Healthy Nevada 102 in 26 906, NorthShore 29 in 9797, Japan 23 of 215 participants. Very few individuals knew about their genetic risk prior to return from one of these programs: Mt. Sinai 1 in 8, Geisinger 0 in 93, Healthy Nevada 3 in 102, and NorthShore and Japan not reported.

### Create new clinical teams

Articles reporting creation of a multidisciplinary lipid clinic composed of different specialists to improve care of individuals with FH showed this approach to be effective. One clinic found high levels of uptake in genetic counseling and subsequent testing for FH (25% with a genetic risk result for FH (6/24)), and intensification of lipid-lowering therapy that resulted in a 79 mg/dl reduction in average LDL-C (*n* = 12, *P* < 0.001) and 75% (9/12) achieving LDL-C target goals [35^▪▪^]. Another lipid clinic study utilized the SB Criteria to identify individuals with definite and possible FH and found that 100% of patients with definite FH and 25% (34/134) of those with possible FH had a genetic risk variant [36].

### Facilitate relay of clinical data to providers

Clinical data that is imperative to the care of individuals with FH should be communicated quickly and in a way that is usable by providers. Clinical decision support tools can be used to prompt providers to identify and treat individuals; however, information on the format, placement, content, timing and frequency, and level of alert urgency/prioritization is key to their uptake [37^▪▪^]. Once prompted, clinicians should be familiar with the different risk scores used to predict cardiovascular disease including a genetic risk score [38] and risk models [39,40]. A genetic risk score was found to be associated with increased odds of cardiovascular disease (variant positive odd ratio [OR] = 3.3; 95% CI 1.3–8.2 and variant negative OR = 1.8; 95% CI 1.0–3.3) [38]. A clinical risk model was found to have fair fit in primary (C-statistic: 0.71; 95% CI: 0.68–0.75) and secondary prevention (0.65; 95% CI 0.60–0.70) patients [39]. When including coronary artery calcium scoring to a traditional risk model there was significantly improved prediction of cardiovascular disease (AUC 0.884, 95% CI 0.871−0.894 compared to 0.793, 95% CI 0.779–0.818) [40].

### Involve patients and family members

Strategies to involve patients and family members in the care process are important. The Netherlands implemented a large cascade screening program for family members of individuals who presented to lipid clinics throughout the country. Several publications highlight the success of this government-sponsored program in identifying family members with FH [41]. Norway has implemented the second most successful cascade screening program [42].

Belgium initiated a national pilot project for cascade screening by recruiting probands with DLCN scores ≥6 from specialty care and then visiting their relatives to collect relevant clinical data and obtain a sample for genetic testing [43]. In this study, the FH diagnosis was made either via DLCN or MEDPED and they found 127 probands with FH and subsequently screened 156 relatives [43]. New Zealand implemented a direct contact approach by hosting a hui, a social gathering, that was organized to inform extended family members about the proband's genetic risk variant that included doctors and nurses from a local health practice, extended family members, and elders to discuss how to best manage and access testing and treatment [44]. A closed Facebook group was initiated that housed the family tree (of consented individuals) and offered information to relatives including a family letter for relatives to show their healthcare providers and information about testing and treatment [44]. This approach reached 17 family members from one family [44]. In the United Kingdom, one study tested a 1-h family-based appointment followed up with telephone calls [45^▪▪^]. This intervention found minimal impact on physical activity but improvements in cardiovascular disease risk factors including reduction in LDL-C [46].

The United States has initiated a few pilot cascade screening programs. The FH Foundation recruited CASCADE FH registry participants who did not previously have genetic testing via the patient portal to obtain free genetic testing [47]. Of the 435 eligible, 110 underwent genetic testing, the majority were female, White, with a median age of 52 years [47]. Sixty-four had a positive genetic test for the familial variant and only three relatives consented to undergoing genetic testing [47]. Another study consented individuals to receive genetic testing for FH by evaluating cholesterol results from a web-based risk assessment service (MeTree [48]) linked to EHR information or EHR query alone to identify probands and then confirmed personal or family history of early coronary artery disease without previous genetic testing [49]. Of the 106 probands that met criteria, 53 underwent genetic testing and two had positive results [49]. The two positive probands gave 4 relatives information and subsequently underwent genetic testing with two having positive results [49]. Motivational interviewing and the extended parallel process model with probands has been tested as an intervention to improve cascade screening and found on average 2.23 new relatives were contacted and 2.46 were screened [50]. A feasibility study based on core principles from the Dutch model, found that when the FH Foundation served as the agency to directly engage with 11 FH probands, they were able to engage nine relatives [51].

## CONCLUSION

The categorization of the studies in this review of implementation strategies from the ERIC compilation provided a framework for organizing current strategies to improve FH care. Strategies described in this review have been shown to improve identification and adherence to guideline recommendations for individuals with FH. Included studies were only mapped to 5 of the 73 implementation strategies from ERIC compilation. This identifies potential areas for research and development of implementation strategies to target unaddressed aspects to improve FH care. In addition, only 8 of the 37 studies included utilized an implementation science theory, model, or framework and only two addressed health disparities and equity in FH care. Application of implementation science and categorization of strategies are important to understanding their benefit and tailoring future strategies to improve care for any cardiovascular condition.

## Acknowledgements

*Katrina Romagnoli, MLIS, PhD, medical librarian, for assisting with the literature search.*

### Financial support and sponsorship

*Research reported in this publication was supported by the National Heart, Lung, and Blood Institute (number K12HL137942, the National Cancer Institute (number P50CA244431), the National Institute of Diabetes and Digestive and Kidney Diseases (numbers P30DK092950 and R25DK123008), the Centers for Disease Control and Prevention (number U48DP006395). The findings and conclusions in this paper are those of the authors and do not necessarily represent the official positions of the National Institutes of Health or the Centers for Disease Control and Prevention.*

### Conflicts of interest

*There are no conflicts of interest.*

## REFERENCES AND RECOMMENDED READING

Papers of particular interest, published within the annual period of review, have been highlighted as:

▪ of special interest
▪▪ of outstanding interest
